# Virtual Reality and Immersive Environments on Sensory Perception of Chocolate Products: A Preliminary Study

**DOI:** 10.3390/foods9040515

**Published:** 2020-04-20

**Authors:** Yanzhuo Kong, Chetan Sharma, Madhuri Kanala, Mishika Thakur, Lu Li, Dayao Xu, Roland Harrison, Damir D. Torrico

**Affiliations:** Department of Wine, Food and Molecular Biosciences, Lincoln University, Lincoln 7647, New Zealand; yanzhuokong@outlook.com (Y.K.); chetan.sharma@lincoln.ac.nz (C.S.); Madhuri.KanalaManjunath@lincolnuni.ac.nz (M.K.); Mishika.Thakur@lincolnuni.ac.nz (M.T.); Lu.Li2@lincolnuni.ac.nz (L.L.); Dayao.Xu@lincolnuni.ac.nz (D.X.); Roland.Harrison@lincoln.ac.nz (R.H.)

**Keywords:** virtual reality, immersive environments, acceptability, emotions, chocolate products

## Abstract

Traditional booths where sensory evaluation usually takes place are highly controlled and therefore have limited ecological validity. Since virtual reality (VR) is substantially interactive and engaging, it has the potential to be applied in sensory science. In this preliminary study, three chocolate types (milk, white, and dark) were evaluated under three contextual settings, including sensory booths (control) and two VR environments (360-degree videos using VR headsets: (i) a pleasant sightseeing tour, and (ii) a live music concert). Untrained participants (*n* = 67) were asked to rate their liking and the intensity of different chocolate attributes based on the 9-point hedonic scale and just-about-right-scale (JAR). Emotions were evaluated using the check-all-that-apply (CATA) method. Results showed that there were no significant effects of context type on the tasting experience; however, there were significant effects of chocolate type. Milk and white chocolates were preferred over dark chocolate irrespective of the context type. Additionally, more positive emotions were elicited for the dark chocolate in the “virtual live concert” environment. Dark chocolate under the other two environments was associated with negative emotional terms, such as “bored” and “worried.” In terms of more reliable and ecologically valid sensory responses, further research is needed to match suitable VR environments to different chocolate types.

## 1. Introduction

Virtual reality (VR) is an emerging technology that provides artificially simulated environments based on computer technology and relevant software [1]. These artificial environments can be either recorded videos, pictures, or animated scenes, which are either immersive [2] or non-immersive [3], and either similar or completely different from the real world [4]. In general, both immersive and non-immersive VR environments can be achieved by some common methods, such as the wall projection [2]. However, fully immersive VR environments are usually achieved by VR headsets nowadays. Based on the high-resolution 360-degree vision and 3D sound, VR headsets provide users with a highly interactive and engaging experience by mobilizing their sight and hearing [5].

VR has become increasingly common in our daily life. For example, VR can enrich people’s entertainment life on the basis of immersive sensory experience regarding games, movies, travelling, and even shopping [6]. It has also been commonly applied in training and education areas, such as supporting the teaching and learning process [7]. A few studies have also explored its application in medicine and tourism [8,9]. Apart from that, applying VR technologies in sensory science is an emerging research field due to the limited ecological validity of traditional sensory booths [10].

The eating environment is considered to have a significant impact on consumers’ sensory perception as well as hedonic responses towards most food products [10]. In sensory science, booths can be used for the sensory evaluation of food products. However, the sensory responses obtained under booths may not describe the totality of the eating experience since the testing environment is highly controlled [10]. This could be one of the reasons why many new products, which were the most liked in consumer trials, eventually failed after launching into the marketplace [11]. The highly controlled sensory booths do not reflect the real consumption environment, and eventually, the obtained results from such setups could have a low ecological validity [12]. However, it seems challenging and relatively expensive to evaluate food products in various real environments. Therefore, the interest in using VR technologies in sensory science has increased dramatically in recent years.

The application of VR technologies in sensory science helps us better understand ecologically valid consumer experiences for certain food products [13]. A few studies have applied VR technologies in sensory science by simulating physically immersive environments, such as a bar, a coffeehouse, and an airplane [14,15,16]. In general, the perceived appropriateness and enjoyment of the eating process for certain food products could be largely influenced by the context. Sinesio et al. [2] observed higher liking scores of tomato and wild rocket salads when they were tasted in an immersive multisensory room compared to the traditional sensory booths. However, the discrimination efficacy of freshness of both vegetables was lower in the immersive environment than in the booths. As different food categories could elicit different sensory perceptions from participants, it is also necessary to take food categories into consideration. For example, Picket and Dando [17] tested how context influences the sensory perception of two alcoholic drinks, beer and sparkling wine. The results showed that the suitable and matched environments for each alcoholic drink tended to improve participants’ acceptance and willingness to purchase. 

As a common snack, chocolate tends to elicit more emotions than other snacks, such as chips [18]. Therefore, three major chocolate products, which were white chocolate, milk chocolate, and dark chocolate, have been evaluated in this preliminary research to better understand consumers’ sensory acceptability and emotional responses affected by immersive VR environments. Three contextual settings were applied in the sensory evaluation process, including two 360-degree recorded videos using VR headsets, and traditional sensory booths as the control setting. As there is an increasing awareness of limited ecological validity in sensory tests, this study also aimed to preliminarily explore the potential of VR technology as a support in regular sensory tests.

## 2. Materials and Methods

### 2.1. Participants

A total of 67 untrained participants (31 males and 36 females, 20–50 years old, originally from Asia, South America, Oceania, and Europe) were recruited voluntarily through Lincoln University intranet. All participants claimed that they were not allergic to the chocolate ingredients involved in this research. A brief introduction about products and sensory procedures was given first. According to Lincoln University Policies and Procedures, they were asked to complete the consent form regarding human ethics (approval: 2019-68) before tasting the chocolate products. Sensory sessions were carried out at the sensory laboratory located in the Replacement For Hilgendorf (RFH) building, Lincoln University, Lincoln, New Zealand. There were three sessions conducted on four consecutive days: one control session using sensory booths and two sessions using VR settings. The duration of each session was around 15 to 25 min per participant, and the order of the three sessions was randomized for each participant. Participants were asked to refrain from eating, drinking, and smoking for at least one hour before the sessions.

### 2.2. Stimuli

Three major chocolate types were used in this research, including Whittaker’s 28% Cocoa White Chocolate, Whittaker’s 33% Cocoa Creamy Milk Chocolate, and Whittaker’s 72% Cocoa Dark Ghana Chocolate (J.H. Whittaker and Sons, Ltd., Porirua, New Zealand). These chocolate products were purchased from a local supermarket before conducting sensory sessions. They were purchased in blocks and served in squares, and they were stored in sealed containers at 4 °C in a refrigerator (Samsung, Seoul, South Korea) when they were not in use. The preparation and sampling processes were conducted within two hours prior to the sensory sessions to prevent chocolate samples from being stale. Three stimuli (white, milk, and dark chocolate) were assessed initially by a focus group panel (*n* = 4) within Lincoln university to make sure they had notable differences in terms of certain attributes, such as sweetness and cocoa flavor. In each sensory session, each chocolate square was served in a transparent plastic cup coded with a 3-digit random number for identification. The presentation order of the three samples was randomized and balanced for each participant to prevent positional bias.

### 2.3. Sensory Procedure

At the beginning of sensory sessions, a brief explanation of the procedures was given to all participants. They were instructed regarding the proper operation and wearing of the VR headsets as well as how to answer questions in tablets (Galaxy Tab A, Samsung, Seoul, South Korea). Three sensory sessions were randomly carried out for each participant. After signing the consent form, participants were instructed to evaluate three randomly ordered chocolate samples from left to right under one of three contextual settings (booth or two VR settings). In the questionnaire, participants were asked to rate the acceptability of ten attributes of each chocolate sample, including taste/flavor, sweetness, bitterness, cocoa flavor, dairy flavor, texture, hardness, smoothness, aftertaste, and overall liking. The acceptability test was based on the 9-point hedonic scale, which represented nine hedonic responses using number 1 (dislike extremely) to 9 (like extremely) with a neutral response at 5 (neither like nor dislike) [19]. Sweetness, bitterness, cocoa flavor, dairy flavor, and overall texture were also evaluated by a just-about-right-scale (JAR) in terms of both intensity and acceptability (1 = too little, 2 = just about right, 3 = too much for sweetness, bitterness, cocoa flavor, and dairy flavor; 1 = too soft, 2 = just about right, 3 = too hard for overall texture) [20].The next question was purchase intent of each chocolate sample (Would you purchase this product if it was available at a reasonable price where you normally shop?), and the answer was based on a binomial scale (1 = No, 2 = Yes). The last section of this questionnaire was the evaluation of emotional responses (Please select all the emotions that you think apply regarding this chocolate sample). The check-all-that-apply (CATA) method was used with a list of 33 emotional terms that were pre-selected previously from 48 original terms [21,22]. These terms were “adventurous,” “satisfied,” “active,” “affectionate,” “calm,” “energetic,” “enthusiastic,” “free,” “friendly,” “glad,” “good,” “happy,” “interested,” “joyful,” “loving,” “merry,” “nostalgic,” “peaceful,” “pleased,” “pleasant,” “secure,” “warm,” “bored,” “disgusted,” “worried,” “aggressive,” “daring,” “eager,” “guilty,” “polite,” “steady,” “understanding” and “wild.” Plain crackers and water were used to cleanse participants’ palate in between different chocolate samples.

### 2.4. Contextual Settings

**Figure 1:**
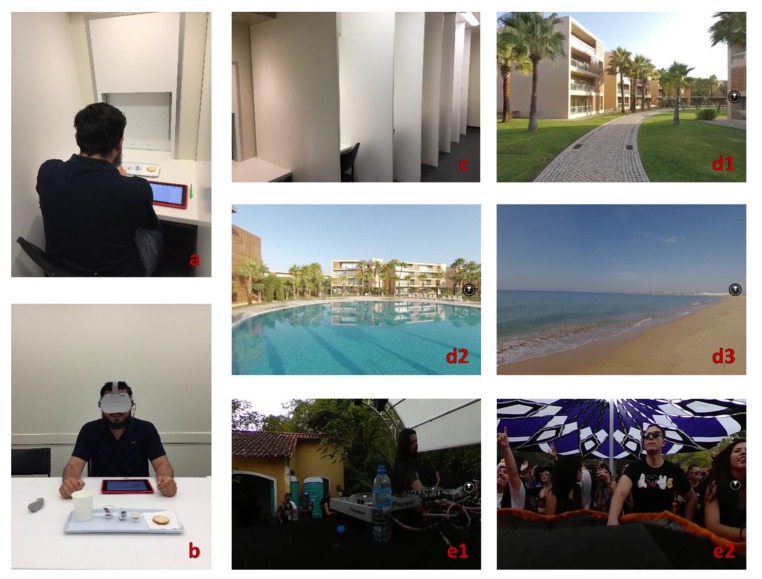
Contextual settings for the sensory evaluation of chocolate products. (**a**) Booths set up; (**b**) Virtual reality (VR) set up; (**c**) Sensory booth setting; (**d**, **1**–**3**) Positive VR setting; (**e**, **1**–**2**) Negative VR setting. VR environments were obtained from the VeeR VR app (VeeR VR, San Francisco, CA, USA).

Three contextual settings were used in this research, including the traditional sensory booth as the control setting, and two VR settings achieved by VR headsets (Oculus VR, LLC., Menlo Park, CA, USA). The sensory booth was located at the RFH building, Lincoln University, Lincoln, New Zealand. Individual booth units were separated by a solid protection panel, and there was a worktop within each isolated booth unit for placing samples and tablets (Figure 1a,c). The booth temperature was set to 21 °C, and white-color fluorescent lights were used during the entire sensory evaluation process.

For sensory sessions associated with the two VR settings, they were carried out in an isolated focus room, which differed from the booth (Figure 1b). Four Oculus Go All-in-One VR Headsets (32 GB) with independent controllers were used for the generation of immersive VR environments. Both VR environments were 360-degree videos selected from a video, movie, and photo platform called VeeR VR, which was available within the VR headset itself (VeeR VR, San Francisco, CA, USA). VeeR VR is a premium VR entertainment platform with more than ten thousand high-quality videos, photos, and interactive experiences. The VR settings used for this research were selected by a focus group (*n* = 4) from a list of 14 preliminarily evaluated 360-degree videos (VeeR VR). The criterion for selecting the videos was based on the experienced pleasantness that participants had during the test in the focus group session. One pleasant and one unpleasant video were selected for further analysis. There were at least two instructors to help participants wear the VR headsets and place samples during the entire sensory session under the VR settings. Chocolate samples were served to participants after they were wearing the headsets and earphones. Participants were instructed to take the headsets off and start answering questions on the tablets when they finished the tasting. This process was repeated until the tasting of all three chocolate samples has been completed for each participant under each VR setting.

With regard to the VR environments, two 360-degree recorded videos (VeeR VR) that could elicit different feelings and emotional responses from participants were selected. The first VR environment was titled as “Pure relaxation in the luxurious apartments Vidamar resorts Algarve.” As shown in Figure 1d1–d3, this video was a sightseeing tour in a 5-star hotel located in Guia, Portugal. It had a duration of 41 min along with relaxing music, which showed beautiful sceneries such as a large swimming pool and a peaceful beach. The second VR environment was titled “Elemental Live—Halloween” (Figure 1e1,e2), which had a duration of 54 min. It was a noisy live music concert, which was held on a cloudy day and was crowded with people. The first and second VR environments were represented by the labels positive VR (PVR) and negative VR (NVR) respectively for convenience, and B was used to represent traditional sensory booths as well.

### 2.5. Statistical Analysis

A 3 × 3 factorial design was used in this research, which referred to three chocolate products and three contextual settings. The acceptability data for sensory attributes were tested for normality by the Shapiro–Wilk test procedure and all attributes that were found violating the normal distribution were transformed through the PROC RANK procedure of Statistical Analysis System-SAS (SAS, Cary, NC, USA) for the subsequent Friedman’s test (RStudio, Version 1.1.456—© 2009-2018 RStudio, Inc.). To assess whether the reported sensory differences among chocolate type × environment were significant or not, we used the non-parametric Friedman and post-hoc Nemenyi tests. The Friedman test first establishes whether at least one of the treatments is significantly different from the rest, and if this was the case, we used the Nemenyi test to identify those treatments for which there is evidence of statistically significant differences. Nememyi test can be used [23,24], provided that data has an equal sample size between each group and Friedman-type ranking of the data. The advantage of this testing approach is that it does not impose any distributional assumptions and does not require multiple pairwise testing between treatments, which would distort the outcome of the tests. The package *tsutils* for RStudio implications were used to run the Nemenyi test (R package version 0.9.2.) [25]. Penalty analysis was applied to the JAR data in order to determine how much the overall liking and acceptance of the chocolate samples were influenced by their attributes. The effect of contextual settings was also considered in this research. The CATA emotional responses of chocolate samples under different settings were assessed by the Cochran’s Q test, Correspondence analysis (CA), and principal coordinate analysis (PCoA) [22,26]. With regard to the purchase intent, it was analyzed for multiple comparisons based on Cochran’s Q test and the simultaneous confidence intervals testing as well. Principal component analysis (PCA; Correlation Biplot) was used to analyze the relationship between the hedonic acceptability of ten attributes and chocolate samples under different environmental settings. The PCA results are presented as a product-attribute biplot. Chocolate samples under different settings were categorized by the Agglomerative Hierarchical Cluster (AHC) analysis. The dissimilarity of them was analyzed based on the Euclidean distance and the Ward’s method. The responses given through an electronic questionnaire were collected by RedJade Sensory Software (Martinez, CA, USA). Data were analyzed using Minitab 18 (Minitab, LLC, State College, PA, USA) and XLSTAT Statistical Software 2016 (Addinsoft, New York, NY, USA). 

## 3. Results

### 3.1. The Effect of Environments on Sensory Acceptability of Chocolate Products

#### 3.1.1. Hedonic Ratings

The sensory attributes measured on 9-point interval scale were analyzed under the null hypothesis that all treatment groups were taken from populations with the same median, and they do not evoke different liking in consumers. Friedman’s two-way nonparametric analysis of variance (ANOVA) was used to evaluate the effect of context environment and chocolate type on the response. By using the ANOVA procedure in conjunction with the PROC RANK procedure, data was ranked ‘not’ by block to analyze the effect of context, whereas traditional method of rank assigning was used, i.e., rank within blocks for the chocolate type. As Friedman’s test cannot be used for interaction, another method was used, where the non-normal distributions were transformed by NORMAL = BLOM function of PROC RANK [27]. This function computes normal scores from the ranks using the formula yi = (1/P) (ri-3/8)/(*n* + 1/4), and after normalization the data were evaluated by using PROC GLM (general linear model) procedure for main and interaction effects. Each response was grouped by two categorical factors, namely context type, and chocolate type, and ranks were assigned by PROC RANK in SAS. In a nutshell, the chocolate type effect was statistically significant, whereas the environment effect was not significant at 95% confidence level. Furthermore, the interaction between chocolate types and environments did not significantly affect the liking scores of the evaluated sensory attributes at 95% confidence. Interestingly, the cocoa flavor was affected by the environment effect at about 88% confidence level.

**Figure 2:**
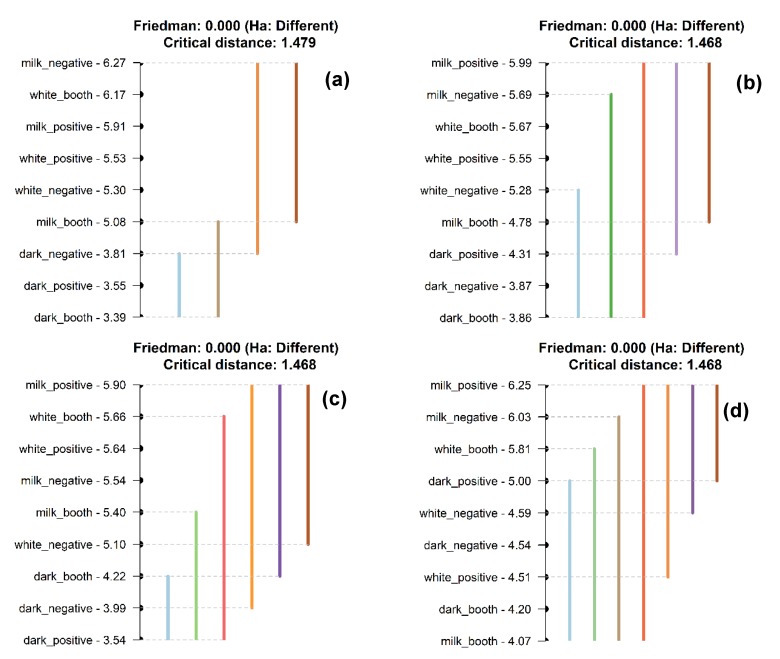

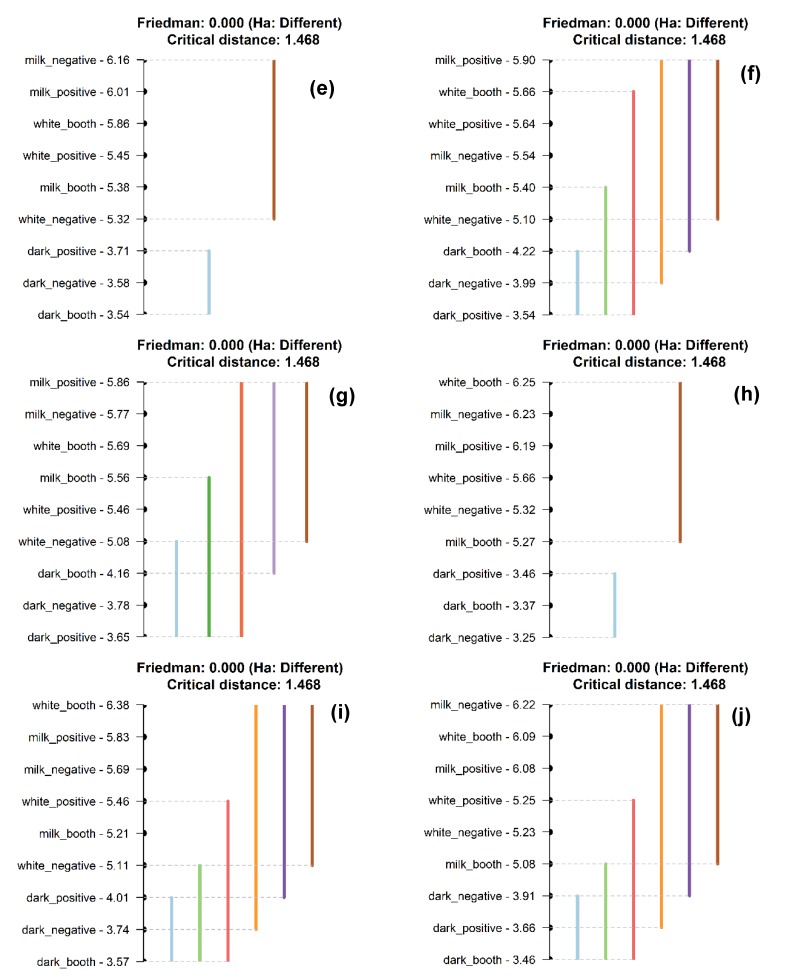
Nemenyi test (post hoc) results comparing medians at 5% significance level for sensory attributes, (**a**) taste, (**b**) sweetness, (**c**) bitterness, (**d**) cocoa flavor, (**e**) dairy flavor, (**f**) texture, (**g**) hardness, (**h**) smoothness, (**i**) aftertaste, and (**j**) overall. Treatments sharing color implies not statistically different.

Consumers that tasted the different chocolate types showed a significant difference in the liking of sensory attributes and thus, accepting the alternate hypothesis that all treatments groups do not belong to the same population median. Nemenyi test, similar to Tukey-HSD (honestly significant difference), helped identify the treatments responsible for the significance of the chocolate effect by comparing the medians of distribution (Figure 2). In general, milk chocolate had the highest liking scores of the evaluated sensory attributes, followed by white chocolate. However, the two types of chocolate products (milk and white) were not significantly different (sharing the same color, brown line) regarding the liking scores of most attributes except for cocoa flavor (Figure 2d). Milk chocolate under PVR and white chocolate under B scored 6.51 ± 0.20 and 5.06 ± 0.24, respectively, for the liking of cocoa flavor, which were significantly different from each other. Dark chocolate was the least liked chocolate type (aqua color line), and its liking scores of taste/flavor, dairy flavor, texture, smoothness, aftertaste, and overall liking were significantly different from other two chocolate types regardless of the environments. Considering the effect of environments, there were no significant differences among PVR, NVR, and B within the same type of chocolate product (*p* > 0.05). The liking scores of the evaluated attributes under PVR were similar and generally high (but not significant, *p* > 0.05) for both milk chocolate and white chocolate, whereas generally high (but not significant, *p* > 0.05) liking scores of evaluated attributes were obtained under NVR for dark chocolate.

#### 3.1.2. Just-about-Right (JAR) Results

**Figure 3:**
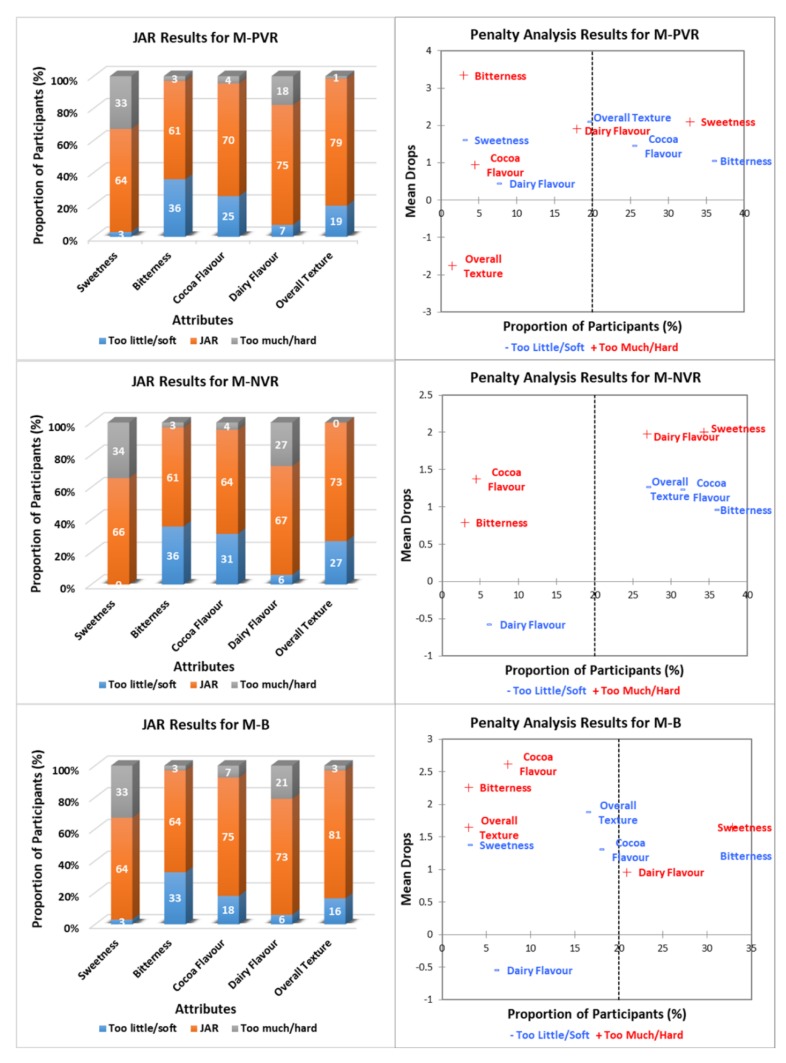
Just-About-Right (JAR) frequencies and penalty analysis results ^1^ regarding milk chocolate attributes under different environments ^2^. ^1^ Penalty analysis was associated with the overall liking scores (9-point hedonic scale). ^2^ M-PVR: milk chocolate positive VR; M-NVR: milk chocolate negative VR; M-B: milk chocolate sensory booth.

**Figure 4:**
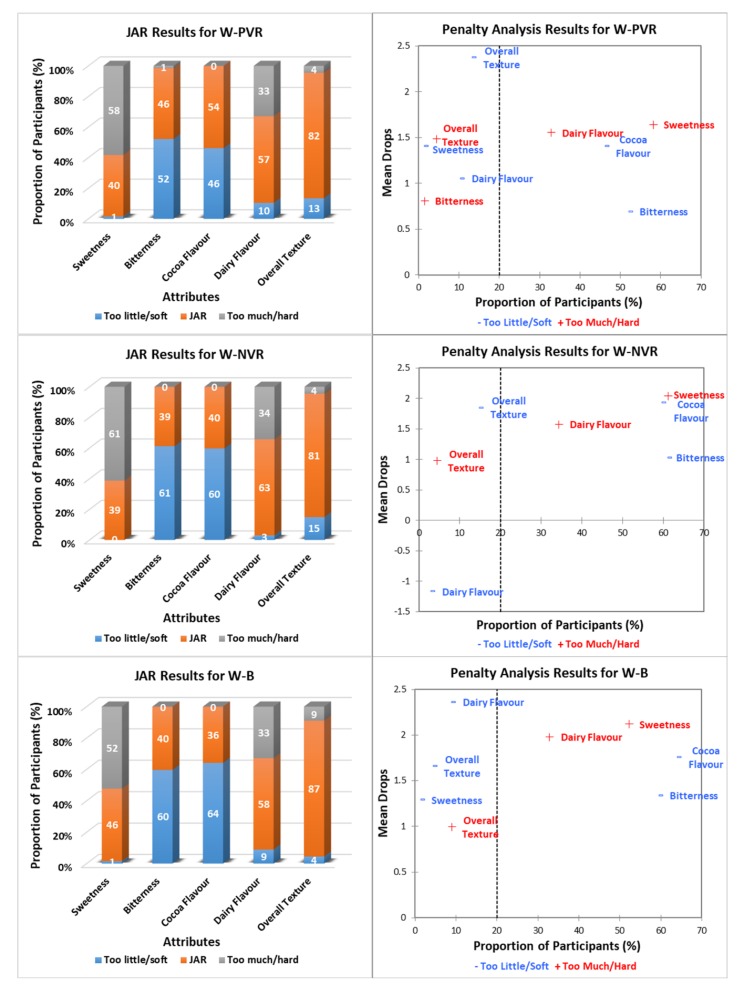
Just-About-Right (JAR) frequencies and penalty analysis results ^1^ regarding white chocolate attributes under different environments ^2^. ^1^ Penalty analysis was associated with the overall liking scores (9-point hedonic scale). ^2^ W-PVR: white chocolate positive VR; W-NVR: white chocolate negative VR; W-B: white chocolate sensory booth.

**Figure 5:**
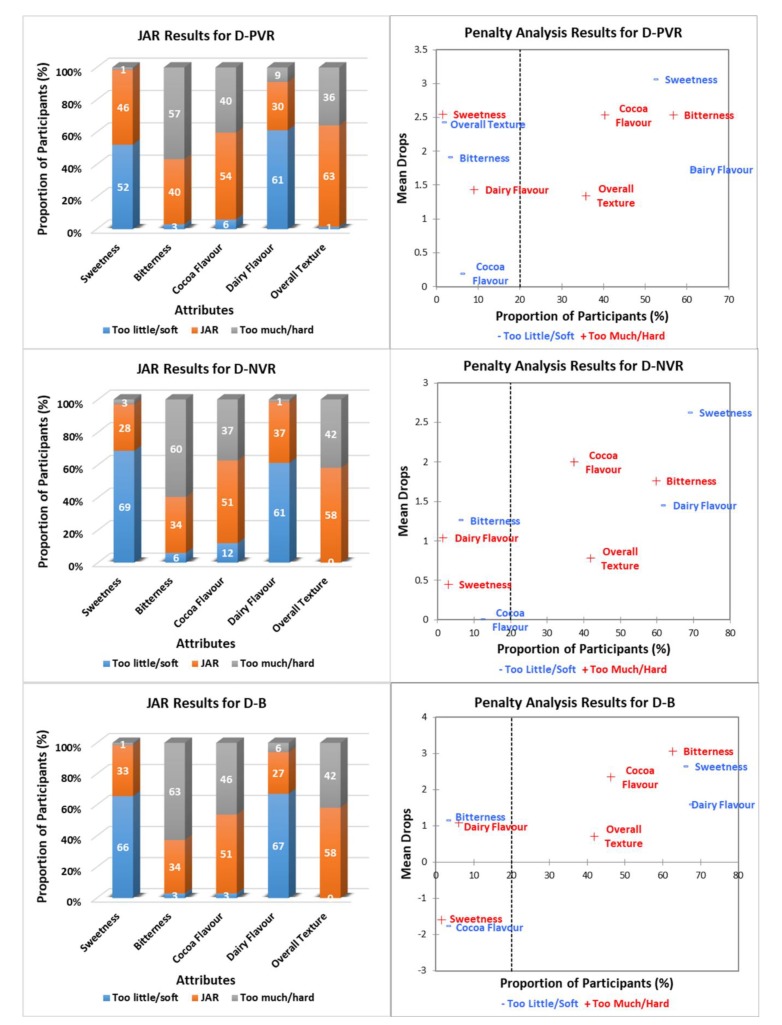
Just-About-Right (JAR) frequencies and penalty analysis results ^1^ regarding dark chocolate attributes under different environments ^2^. ^1^ Penalty analysis was associated with the overall liking scores (9-point hedonic scale). ^2^ D-PVR: dark chocolate positive VR; D-NVR: dark chocolate negative VR; D-B: dark chocolate sensory booth.

Figure 3, Figure 4 and Figure 5 show the JAR frequencies (%) and mean drops based on the penalty analysis for the three chocolate products (milk, white, and dark) under three environments (PVR, NVR, and B), regarding their sweetness, bitterness, cocoa flavor, dairy flavor, and overall texture. As shown in Figure 3, milk chocolate had the highest selections of JAR for cocoa flavor (75%) and overall texture (81%) under B, whereas the highest proportion of participants (75%) selected JAR for dairy flavor under PVR. Milk chocolate under NVR had the highest selections of “too little/soft” for both cocoa flavor (31%) and overall texture (27%), and “too much” for dairy flavor (27%). The frequencies of sweetness and bitterness for milk chocolate were similar under three environments. For white chocolate (Figure 4), JAR was selected most frequently for sweetness (46%) and overall texture (87%) under B, as well as for bitterness (46%) and cocoa flavor (54%) under PVR. White chocolate under NVR had the highest selection of JAR for dairy flavor (63%). Selections of “too much/hard” for bitterness (0–1%), cocoa flavor (0%) and overall texture (4–9%), as well as “too little” for sweetness (0–1%) and dairy flavor (3–10%) of white chocolate were negligible regardless of the environments. With regard to dark chocolate (Figure 5), JAR was selected most frequently for sweetness (46%), bitterness (40%), cocoa flavor (54%), and overall texture (63%) under PVR. Dark chocolate under NVR had the highest selection of JAR for dairy flavor (37%). The frequencies of “too much” regarding sweetness, dairy flavor, and “too little/soft” regarding bitterness, cocoa flavor, the overall texture of dark chocolate under three environments were negligible.

Penalty analysis was conducted based on both JAR frequencies and the overall liking scores of chocolate products considering the environments. As shown in Figure 3, Figure 4 and Figure 5, the threshold for the population size was set as 20%. The attributes that appeared in the upper right-hand corner of the penalty plot were considered to have negative effects on the liking of products, as more than 20% of people thought they were either “too much/hard” or “too little/soft” [28]. Accordingly, both milk and white chocolate were penalized for being too sweet and not bitter enough, which was opposite to dark chocolate. Milk chocolate under both PVR and NVR was penalized due to not having enough cocoa flavor. In addition, “too much” dairy flavor for milk chocolate under both NVR and B also affected their overall liking scores. The penalty analysis results for both white chocolate and dark chocolate were generally consistent under the three tested environments, of which cocoa flavor and dairy flavor were penalized for being “too little” and “too much” for white chocolate, respectively, whereas the opposite happened with dark chocolate. Moreover, the liking of dark chocolate was affected by its hard texture as well (Figure 5).

### 3.2. Multivariate Analysis of Chocolate Products under Different Environments

#### 3.2.1. Emotional Responses

**Figure 6:**
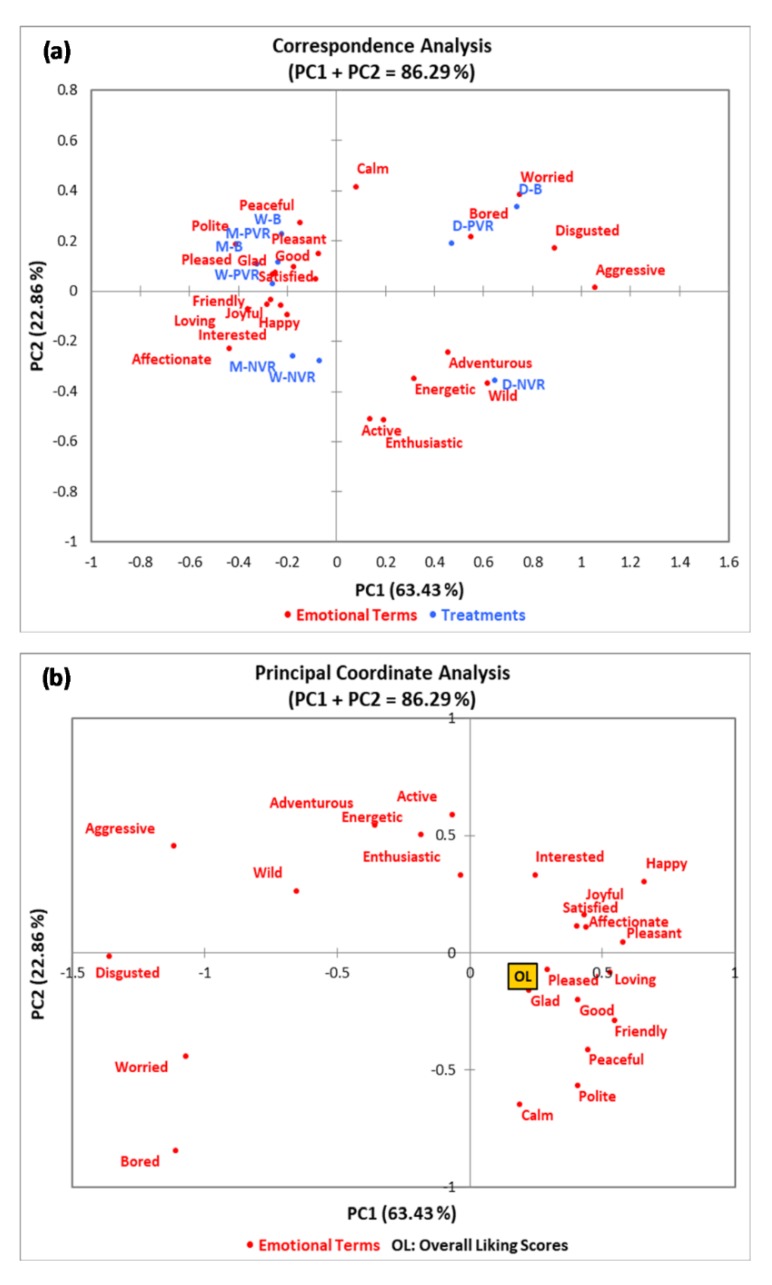
(**a**) Correspondence analysis (CA) of emotional terms for chocolate products tasted under different contextual settings ^1^; (**b**) Principal coordinate analysis (PCoA) of emotional terms regarding the overall liking scores. ^1^ M-PVR: milk chocolate positive VR; M-NVR: milk chocolate negative VR; M-B: milk chocolate sensory booth; W-PVR: white chocolate positive VR; W-NVR: white chocolate negative VR; W-B: white chocolate sensory booth; D-PVR: dark chocolate positive VR; D-NVR: dark chocolate negative VR; D-B: dark chocolate sensory booth.

According to Cochran’s Q test results (Table S1), 23 emotional terms were significant, including “adventurous,” “satisfied,” “active,” “calm,” “affectionate,” “energetic,” “enthusiastic,” “friendly,” “glad,” “good,” “happy,” “interested,” “joyful,” “loving,” “peaceful,” “pleased,” “pleasant,” “bored,” “disgusted,” “worried,” “aggressive,” “polite”, and “wild.” Figure 6 shows the correspondence analysis (CA) and principal coordinate analysis (PCoA) results. The CA displays the relationships between emotional terms obtained based on the CATA method and three chocolate products considering the contextual effect. As shown in Figure 6a, the principal component one (PC1) and principal component two (PC2) were 63.43% and 22.86%, respectively, which explained 86.29% of data variability in total. According to CA results, milk chocolate and white chocolate under PVR and B were found to share similar profiles, which were associated with both neutral and positive emotional descriptors such as “peaceful,” “pleasant,” “good,” “satisfied,” “glad,” “pleased,” and “polite.” Milk and white chocolate under NVR were also associated with positive terms, such as “affectionate,” “interested,” “happy,” “loving,” “joyful,” and “friendly.” Dark chocolate had highly distinctive groups of emotional terms under NVR and PVR/B. With regard to dark chocolate under NVR, it was related to ardent descriptors, including “adventurous,” “energetic,” “wild,” “active,” and “enthusiastic.” In contrast, dark chocolate under PVR and B were related to negative terms, such as “bored,” “worried,” “disgusted,” and “aggressive.” 

The PCoA results show the relationship between emotional terms and the overall liking scores of the three chocolate products under three different contextual settings (Figure 6b). Only the terms “aggressive,” “disgusted,” “worried,” and “bored” were selected in relation to the lowest mean values (<5.0) for the overall liking of chocolate products under different environments. However, terms such as “pleased,” “glad,” “good,” “loving,” “friendly,” “peaceful,” “pleasant,” “affectionate,” “satisfied,” and “joyful” contributed to higher overall liking scores of chocolate products considering the contextual effect (>5.0).

#### 3.2.2. Principal Component and Cluster Analyses of the Chocolate Products under Different Environments

**Figure 7:**
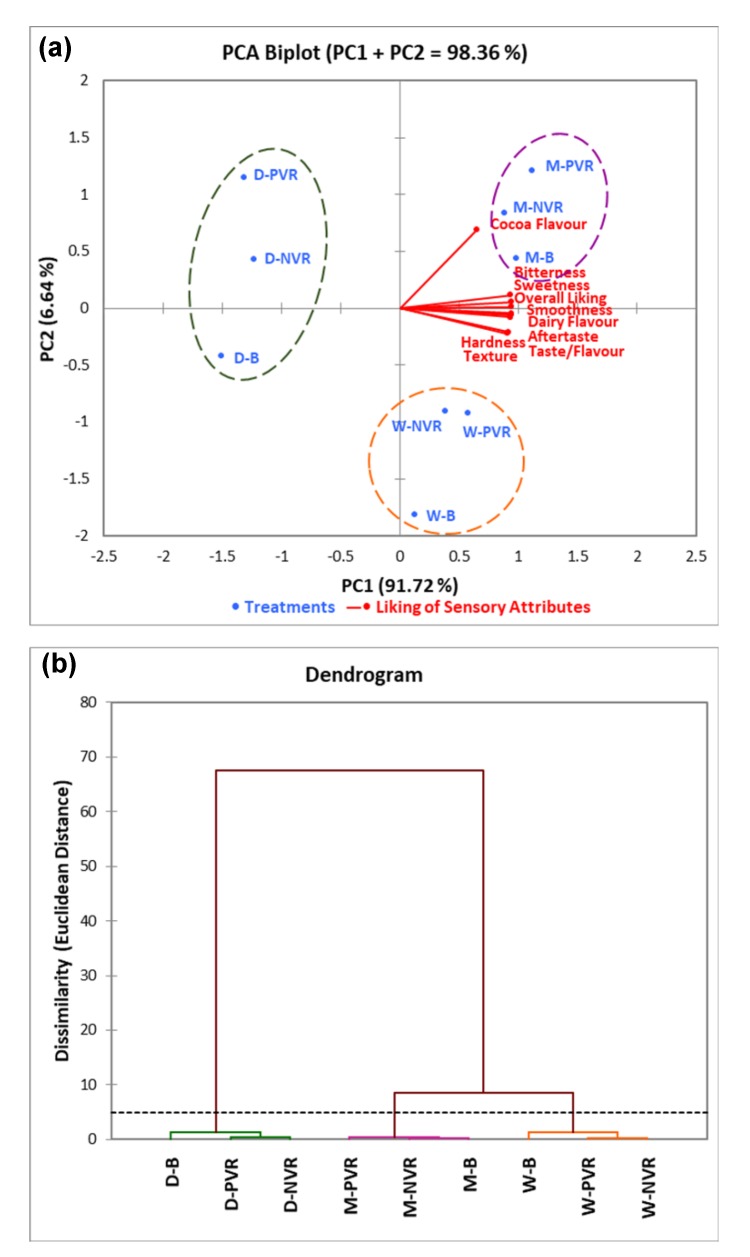
(**a**) Principal component analysis (PCA) biplot regarding liking scores ^1^ of chocolate attributes in different environments ^2^; (**b**) Dendrogram of agglomerative hierarchical clustering (AHC) grouping chocolate products under different environments ^2^. ^1^ Liking scores were based on the 9-point hedonic scale (1 = dislike extremely, 5 = neither like nor dislike, 9 = like extremely) [19]. ^2^ M-PVR: milk chocolate positive VR; M-NVR: milk chocolate negative VR; M-B: milk chocolate sensory booth; W-PVR: white chocolate positive VR; W-NVR: white chocolate negative VR; W-B: white chocolate sensory booth; D-PVR: dark chocolate positive VR; D-NVR: dark chocolate negative VR; D-B: dark chocolate sensory booth.

The principal component analysis (PCA) and agglomerative hierarchical clustering (HCA) results are shown in Figure 7. PCA biplot visualized the associations between liking scores of ten attributes and the three chocolate products (milk, white, and dark) while considering the contextual effect (Figure 7a). The principal component one (PC1) and principal component two (PC2) were 91.72% and 6.64%, respectively, explaining totally 98.36% of data variability. Liking vectors of most attributes were well linked with the horizontal axis, which was PC1 (squared cosines varied from 0.91 to 0.99). The liking vector of cocoa flavor was aligned with the vertical axis, which was PC2 (squared cosine was 0.53). Liking vectors of most attributes, except for cocoa flavor, were close to each other in Figure 7a, indicating their positive association. In addition, the liking vector of cocoa flavor was not associated with the liking vectors of hardness and texture as they were almost orthogonal. In terms of chocolate products, milk chocolate was highly associated with the liking of cocoa flavor under PVR and NVR, and milk chocolate under B was associated with the overall liking as well as the liking of bitterness, sweetness, smoothness, dairy flavor, aftertaste and taste/flavor. In addition, white chocolate was relatively associated with the liking of hardness and texture under PVR and NVR. However, dark chocolate was negatively correlated with the liking of all evaluated attributes regardless of the contextual effects.

Figure 7b shows the dendrogram based on AHC for the nine chocolate–environment combinations (3 × 3 factorial design). Three main cluster groups were formed, which were (1) dark chocolate under all environments, (2) milk chocolate under all environments, and (3) white chocolate under all environments.

### 3.3. The Effect of Environments on the Purchase Intent of Chocolate Products

The frequencies of purchase intent for three chocolate products (milk, white, and dark) under the three environments (PVR, NVR, and B) are shown in Table 1. In general, milk chocolate had the highest purchase intent (64.2–73.1%) followed by white chocolate (56.7–62.7%), and dark chocolate had the lowest purchase intent (31.3–43.3%). Milk chocolate and white chocolate were not significantly different in their purchase intent under the three environments (*p* > 0.05). However, milk chocolate had significantly higher purchase intent than dark chocolate under the PVR, NVR, and B, respectively (*p* < 0.05). Based on the results, participants tended to be more willing to purchase three chocolate products under PVR, whereas there were no significant differences among PVR, NVR, and B regarding each chocolate type (*p* > 0.05).

## 4. Discussion

### 4.1. The Effect of Environments on Sensory Acceptability of Chocolate Products

#### 4.1.1. Hedonic Ratings

The effect of context was found to not significantly affect the tasting experience of the chocolate types. The increased social element (via context) was found here not contributing enough to change the eating experience. Many reasons could be responsible for this finding, such as:

(a) Large product effect with respect to empathy, [29], emotions [29], ecstasy, involvement or indulgent;
(b) Irrelevant consumption context [30];
(c) Familiarity with the product, thus context plays a smaller role [31,32];
(d) Strong preference effect compared to context effect with respect to chocolate type, which may consequently help explain the stable preference pattern for chocolates, namely milk and white chocolate type;
(e) Meal context, not testing context, is the more effective influence [31];
(f) Attention bias could also make consumers focused only on chocolate testing and not to be affected by context environments, as consumers were aware of testing chocolate in three settings.

Pound and Duizer (2000) found similar no context effect results with chocolate type tested in four testing situations, namely, central location, in-home, teaching laboratory, and sensory laboratory. Similarly, context has previously been found to have no effect on the eating experience of cheese [30]. Thus, the effect of context depends on many of the abovementioned factors, which can contribute to the variability of the results.

In the present study, the type of chocolate was found to have significant effects on the hedonic ratings towards certain sensory attributes. Milk chocolate was the most liked product, followed by white chocolate, whereas the dark chocolate was the least liked among all the products (Figure 2). The three types of chocolate products used in this research varied in cocoa content, which were 28% (white), 33% (milk), and 72% (dark). Therefore, their sensory attributes can be largely affected by their ingredients, such as sweetness, bitterness, and cocoa flavor. Glicerina et al. [33] reported that these three chocolate types also have different textual properties. White chocolate has less aggregate structure and the lowest viscosity, whereas dark chocolate has the highest aggregate structure and fewer void spaces between particles. The microstructure and rheological properties of milk chocolate are in-between, which could be the reason for the highest liking scores. It has been reported that consumers prefer less hard and light chocolate products, which could be a support for the findings of the present research as well [34]. Apart from that, consumer preferences and relevant hedonic ratings may also be affected by demographic factors, such as gender [35]. Although the effect of the environment on the hedonic responses was marginal in this study, other reactions of consumers could be affected by the context in which consumers taste the product. As reported by Stelick and Dando [10], the environment where food products are consumed could affect the enjoyment, feelings, and purchase intent of alcoholic drinks [17].

#### 4.1.2. JAR Results

JAR results can describe both the acceptability and intensity towards sensory attributes of products. In general, milk chocolate was the most acceptable product among the three chocolate types since its JAR selections for all attributes were comparatively high (Figure 3). However, the overall liking of milk chocolate was found to be affected by the contextual settings based on the penalty analysis results. The effect of an attribute on overall liking of the product is considered significant when the proportion of participants’ responses to “not JAR” is greater than the commonly used threshold of 20% [36,37]. In the present study, both PVR and NVR led to the higher selection of “too little,” and lower selections of JAR, and “too much” of the cocoa flavor for the milk chocolate (Figure 3). This might be because both PVR and NVR provided a better engagement than the sensory booth did, in which participants might focus more on the virtual experience than the chocolate itself. Bangcuyo et al. [15] also reported that consumers were more engaged in a coffee evaluation session that took place in an immersive virtual coffeehouse rather than in the traditional sensory booth. Therefore, the finding of this research might indicate that sensory evaluation conducted under immersive VR environments could have better engagement and ecological validity than traditional sensory booths.

The white and dark chocolates had lower JAR selections regarding all attributes compared to milk chocolate (Figure 4 and Figure 5). About 81–87% of participants found that the overall texture of white chocolate under the three environments was just about right (Figure 4). Similar to the penalty analysis results, the other four attributes of white chocolate, including sweetness, bitterness, cocoa flavor, and dairy flavor, were penalized for being either “too much” or “too little” regardless of the environments. For the dark chocolate under three environments, all five attributes tended to have great negative effects on its overall liking (Figure 5). On the other hand, the sweetness and dairy flavor of white chocolate could be reduced and its cocoa flavor and bitterness could be increased for increasing consumers’ liking, while on the contrary for dark chocolate. Overall, contextual settings did not affect the penalty analysis results for the white and dark chocolates. As previously mentioned, white chocolate has 28% cocoa content and less aggregate structure, whereas dark chocolate has 72% cocoa content and the highest aggregate structure [33]. In other words, both chocolate types have extreme cocoa content and textual properties, which could have greater effects than contextual settings (Figure 2). Milk chocolate has relatively moderate cocoa content (33%) and textual properties, which could minimize the effect of chocolate itself and enlarge the effect of contextual settings [33]. This is probably why the penalty analysis results for milk chocolate were different under the three environments.

### 4.2. Multivariate Analysis of Chocolate Products under Different Environments

#### 4.2.1. Emotional Responses

In the present study, the most frequently selected emotional terms changed depending on both chocolate types as well as contextual settings (Figure 6). The descriptors selected for both milk chocolate and white chocolate were generally similar under different environments, which were both neutral and positive, such as “polite” and “affectionate.” However, dark chocolate was associated with distinct emotions in different environments. Dark chocolate under NVR tended to have ardent emotional terms such as “adventurous” and “energetic.” On the contrary, dark chocolate under both PVR and B were associated with negative terms, including “bored,” “worried,” “disgusted,” and “aggressive,” which contributed to low overall liking scores of chocolate products (<5.0). Overall, each chocolate product tasted under PVR elicited generally similar emotions as the control setting, namely sensory booths. However, NVR tended to evoke more passionate emotions from participants, especially towards dark chocolate.

Both VR environments used in this study showed their impacts on consumers’ emotional responses towards chocolate products, especially the NVR. Xu et al. [38] reported that the environments where food products are consumed could heavily affect consumers’ emotions. They found significant changes in emotions evoked from subjects when ice cream was consumed under laboratory, café, university study area, and bus stop settings. Apart from the visual effect, the auditory effect involved in this study should also be considered, as the VR environments were based on videos [39]. The liking of the two VR environments was highly subjective as dark chocolate was associated with negative emotions and low overall liking scores under B, which was the control setting, whereas the “virtual live concert” setting positively affected participants’ emotional responses and overall acceptance of dark chocolate. According to the results, consumers might feel more appropriate to consume a dark chocolate rather than milk or white chocolates in a “live concert” environment. 

#### 4.2.2. Principal Component and Cluster Analyses of the Chocolate Products under Different Environments

Three clusters were formed primarily based on the type of chocolate products, whereas contextual settings were found not significant enough to affect the clustering in the present study (Figure 7). In general, milk chocolate was the most liked chocolate, and dark chocolate was the least liked chocolate based on the PCA results, which is similar to the finding in Figure 2. Both PVR and NVR were found to have positive effects on the cocoa flavor liking of milk chocolate and the textual liking of white chocolate. Some previous studies reported that both enjoyment and hedonic responses of food products tended to be higher under immersive VR environments [2,17]. However, those environments were pleasant and suitable for the consumption of relevant food products, such as tasting sparkling wine in a winery as well as vegetables in a holiday farm. Although the two VR environments used in this research belong to different categories, their positive effects towards certain chocolate attributes were similar. Hathaway and Simons [3] reported that the hedonic ratings of cookies obtained under relevant, immersive VR environments were more reliable and discriminating. Therefore, it would be necessary to select a matched tasting environment for each chocolate product in order to obtain reliable hedonic data. These sensory results could be further applied in testing newly developed chocolate products before launching.

### 4.3. The Effect of Environments on the Purchase Intent of Chocolate Products

In the present study, the purchase intent was highly associated with participants’ hedonic responses as well as the PCA results, in which milk chocolate was the most liked chocolate type (Figure 2 and Figure 7). As discussed above, milk chocolate had moderate cocoa content and textual properties, which could be the primary reason for the consumers’ preference [33]. Although participants were most willing to pay for three chocolate products under PVR, there were no significant differences among PVR, NVR, and B within each chocolate type (*p* > 0.05). Apart from the chocolate category, there are many factors that could affect consumers’ willingness to purchase, such as the nutritional value and health claims of products [40]. Gunaratne et al. [26] also reported that the packaging could have effects on consumers’ acceptance, purchase intent, and emotions regarding chocolate products. On the other hand, the consuming context may also contribute to those impacts. As reported by O’Brien and Toms [41], aesthetically pleasant environments could positively influence consumers’ engagement. The highest frequencies of purchase intent achieved under PVR for each chocolate product might be correlated with improved engagement, which might have been perceived by the participants.

## 5. Conclusions

This preliminary study firstly explored the effect of 360-degree immersive videos (based on VR headsets) on the sensory perception of chocolate products. Changing the context environments did not significant affect the hedonic ratings of chocolates. As compared to the traditional sensory booth, participants tended to have better engagement when they tasted chocolate products under both VR environments. Therefore, the data could be more ecologically valid as well as relevant to actual consumers’ experience. In addition, the combination of dark chocolate and “virtual live concert” significantly affected consumers’ hedonic responses and emotions into a positive and passionate direction. However, it is necessary to further match each chocolate type to a suitable consuming environment achieved by VR headsets, for more reliable and ecologically valid sensory responses.

## Figures and Tables

**Table 1:** Purchase intent frequencies of chocolate products under different environments.

Treatments ^1^		Willingness to Purchase (%) ^2^
Chocolate	Environment	
Milk	PVR	73.1 ^a^
	NVR	64.2 ^ab^
	B	70.1 ^a^
White	PVR	62.7 ^ab^
	NVR	61.2 ^ab^
	B	56.7 ^abc^
Dark	PVR	43.3 ^bc^
	NVR	31.3 ^c^
	B	34.3 ^c^

^1^ three types of chocolate products (milk, white and dark) were tested under three contextual settings (PVR: positive VR, NVR: negative VR, and B: sensory booth). ^2^ Cochran’s Q test was used together with Marascuilo procedure for multiple pairwise comparisons (*n* = 67); ^a–c^ Frequencies with different superscripts within the same column indicate significant differences (Cochran’s Q test and Marascuilo procedure, *p* < 0.05).

## Supplementary Materials

The following is available online at https://www.mdpi.com/2304-8158/9/4/515/s1, Table S1: Frequencies and significance of emotional terms based on Cochran’s Q test.
