# Supplementary material for: Virtual Reality and Immersive Environments on Sensory Perception of Chocolate Products: A Preliminary Study
Source: Foods. 2020 Apr 20;9(4):515. doi: 10.3390/foods9040515 (PMC7230827; doi:10.3390/foods9040515)
Supplement: Supplementary file 1 [file foods-09-00515-s001.pdf]

Table S1. Frequencies and significance of emotional terms [1] based on Cochran's Q test [2].

Emotional Terms	Treatments								
	**M-PVR**	**M-NVR**	**M-B**	**W-PVR**	**W-NVR**	**W-B**	**D-PVR**	**D-NVR**	**D-B**
Adventurous [3]	0.06^{a}	0.08^{a}	0.08^{a}	0.06^{a}	0.13^{a}	0.03^{a}	0.15^{a}	0.16^{a}	0.06^{a}
Satisfied	0.24^{a}	0.25^{a}	0.25^{a}	0.22^{a}	0.22^{a}	0.19^{a}	0.08^{a}	0.10^{a}	0.19^{a}
Active	0.12^{ab}	0.24^{a}	0.09^{ab}	0.15^{ab}	0.22^{ab}	0.05^{b}	0.06^{ab}	0.24^{a}	0.06^{ab}
Affectionate	0.12^{abc}	0.13^{ab}	0.12^{abc}	0.15^{a}	0.15^{a}	0.06^{abc}	0.02^{bc}	0.03^{abc}	0^{c}
Calm	0.21^{a}	0.08^{a}	0.25^{a}	0.13^{a}	0.09^{a}	0.18^{a}	0.18^{a}	0.06^{a}	0.19^{a}
Energetic	0.09^{b}	0.19^{ab}	0.10^{b}	0.13^{ab}	0.30^{a}	0.09^{b}	0.13^{ab}	0.27^{ab}	0.13^{ab}
Enthusiastic	0.06^{ab}	0.19^{a}	0.06^{ab}	0.12^{ab}	0.18^{a}	0.02^{b}	0.09^{ab}	0.16^{ab}	0.05^{ab}
Free	0.18^{a}	0.18^{a}	0.13^{a}	0.19^{a}	0.15^{a}	0.09^{a}	0.10^{a}	0.12^{a}	0.06^{a}
Friendly	0.25^{ab}	0.27^{a}	0.27^{a}	0.19^{ab}	0.25^{ab}	0.22^{ab}	0.10^{ab}	0.08^{ab}	0.06^{b}
Glad	0.19^{abc}	0.21^{abc}	0.28^{a}	0.27^{ab}	0.16^{abc}	0.24^{abc}	0.06^{c}	0.09^{bc}	0.09^{bc}
Good	0.43^{a}	0.34^{abc}	0.37^{ab}	0.36^{abc}	0.27^{abc}	0.37^{ab}	0.16^{bc}	0.15^{c}	0.18^{bc}
Happy	0.34^{ab}	0.39^{a}	0.39^{a}	0.42^{a}	0.37^{ab}	0.39^{a}	0.15^{bc}	0.19^{abc}	0.09^{c}
Interested	0.10^{a}	0.18^{a}	0.16^{a}	0.13^{a}	0.16^{a}	0.16^{a}	0.10^{a}	0.08^{a}	0.02^{a}
Joyful	0.42^{a}	0.27^{abcd}	0.34^{abc}	0.36^{ab}	0.39^{a}	0.27^{abcd}	0.12^{cd}	0.13^{bcd}	0.06^{d}
Loving	0.28^{a}	0.24^{ab}	0.22^{abc}	0.24^{ab}	0.22^{abc}	0.19^{abc}	0.05^{bc}	0.08^{bc}	0.03^{c}
Merry	0.09^{a}	0.10^{a}	0.09^{a}	0.15^{a}	0.13^{a}	0.12^{a}	0.08^{a}	0.09^{a}	0.05^{a}
Nostalgic	0.06^{a}	0.06^{a}	0.08^{a}	0.08^{a}	0.06^{a}	0.03^{a}	0.09^{a}	0.06^{a}	0.12^{a}
Peaceful	0.25^{ab}	0.10^{b}	0.21^{ab}	0.31^{a}	0.10^{b}	0.19^{ab}	0.16^{ab}	0.06^{b}	0.10^{b}
Pleased	0.19^{ab}	0.22^{ab}	0.33^{a}	0.24^{ab}	0.21^{ab}	0.25^{ab}	0.10^{b}	0.08^{b}	0.09^{b}
Pleasant	0.36^{a}	0.25^{ab}	0.30^{ab}	0.24^{ab}	0.21^{ab}	0.24^{ab}	0.25^{ab}	0.10^{b}	0.15^{ab}
Secure	0.02^{a}	0.06^{a}	0.06^{a}	0.08^{a}	0.05^{a}	0.09^{a}	0.05^{a}	0.03^{a}	0.08^{a}
Warm	0.15^{a}	0.19^{a}	0.22^{a}	0.16^{a}	0.12^{a}	0.13^{a}	0.09^{a}	0.10^{a}	0.12^{a}
Bored	0.09^{b}	0.09^{b}	0.10^{b}	0.10^{b}	0.10^{b}	0.21^{ab}	0.15^{ab}	0.22^{ab}	0.30^{a}
Disgusted	0.06^{bc}	0.06^{bc}	0.02^{c}	0.06^{bc}	0.09^{abc}	0.08^{abc}	0.22^{ab}	0.19^{ab}	0.24^{a}
Worried	0.10^{ab}	0.03^{b}	0.03^{b}	0.09^{ab}	0.06^{b}	0.09^{ab}	0.18^{ab}	0.13^{ab}	0.25^{a}
Aggressive	0.05^{bc}	0.03^{c}	0.03^{c}	0.03^{c}	0.08^{abc}	0.02^{c}	0.15^{abc}	0.21^{a}	0.19^{ab}
Daring	0.03^{a}	0.05^{a}	0.08^{a}	0.06^{a}	0.03^{a}	0.02^{a}	0.05^{a}	0.08^{a}	0.06^{a}
Eager	0.05^{a}	0.05^{a}	0.03^{a}	0.12^{a}	0.03^{a}	0.03^{a}	0.06^{a}	0.09^{a}	0.09^{a}
Guilty	0.06^{a}	0.05^{a}	0.10^{a}	0.10^{a}	0.05^{a}	0.10^{a}	0.10^{a}	0.06^{a}	0.13^{a}
Polite	0.10^{ab}	0.08^{ab}	0.18^{a}	0.18^{a}	0.10^{ab}	0.12^{ab}	0.03^{b}	0^{b}	0.05^{ab}
Steady	0.08^{a}	0.05^{a}	0.12^{a}	0.09^{a}	0.05^{a}	0.12^{a}	0.09^{a}	0.10^{a}	0.12^{a}
Understanding	0.02^{a}	0.02^{a}	0.06^{a}	0.08^{a}	0.05^{a}	0.05^{a}	0.02^{a}	0.02^{a}	0.05^{a}
Wild	0.06^{b}	0.12^{ab}	0.06^{b}	0.05^{b}	0.12^{ab}	0.03^{b}	0.06^{b}	0.24^{a}	0.13^{ab}

[1] 33 emotional terms were associated with 3 types of chocolate products and 3 contextual settings; M-PVR: milk chocolate-positive VR; M-NVR: milk chocolate-negative VR; M-B: milk chocolate-sensory booth; W-PVR: white chocolate-positive VR; W-NVR: white chocolate-negative VR; W-B: white chocolate-sensory booth; D-PVR: dark chocolate-positive VR; D-NVR: dark chocolate-negative VR; D-B: dark chocolate-sensory booth;

[2] Cochran's Q test was used together with Marascuilo procedure for multiple pairwise comparisons (N = 67);

[3] Significant emotional terms were ***italicised and bolded*** ($p < 0.05$);

[a-d] Results with different superscripts regarding each emotional term in each row indicate significant differences ($p < 0.05$); The value of each treatment ranges from 0 to 1, higher values indicate higher frequencies.
